# Supplementary material for: Reflectometry Reveals Accumulation of Surfactant Impurities at Bare Oil/Water Interfaces
Source: Molecules. 2019 Nov 14;24(22):4113. doi: 10.3390/molecules24224113 (PMC6891303; doi:10.3390/molecules24224113)
Supplement: Supplementary file 1 [file molecules-24-04113-s001.pdf]

Reflectometry reveals accumulation of surfactant impurities at bare oil/water interfaces: Supplementray Information

Ernesto Scoppola, Samantha Micciulla, Lucas Kuhrts, Armando Maestro, Richard Campbell, Oleg Konovalov, Giovanna Fragneto and Emanuel Schneck

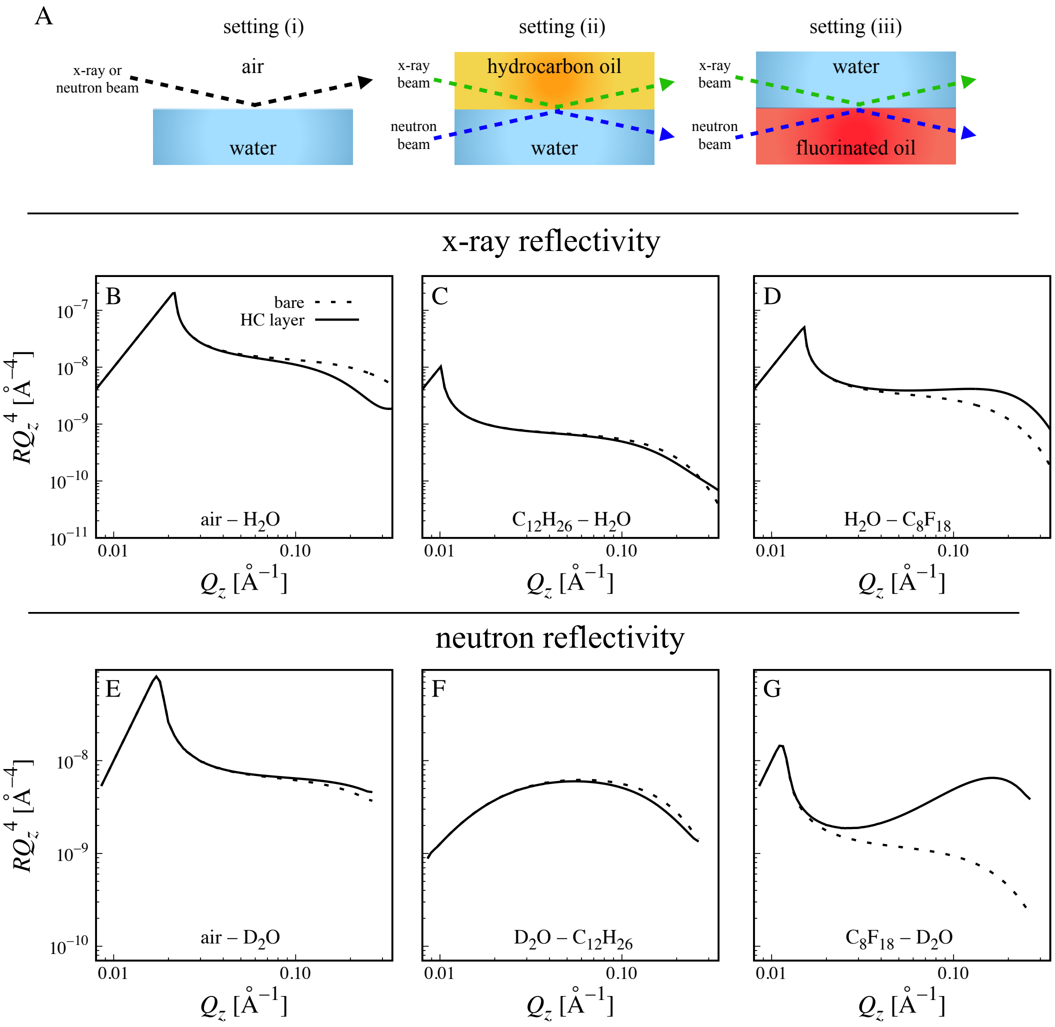

Figure SI.1: Experimental configurations for reflectometry on water/hydrophobic interfaces. (A) Schematic illustrations of (i) water contacting air, (ii) water contacting hydrogenous hydrocarbon oil, and (iii) water contacting fluorinated oil. Arrows indicate the paths of the x-ray or neutron beams, respectively. (B-D) Theoretical x-ray reflectivities based on estimated parameters for configurations (i-iii) with (solid lines) and without (dotted lines) a 10–Å–thick hydrocarbon (HC) layer adsorbed to the interface. (E-G) Theoretical neutron reflectivities for configurations (i-iii) with (solid lines) and without (dotted lines) a 10–Å–thick hydrocarbon (HC) layer adsorbed to the interface.

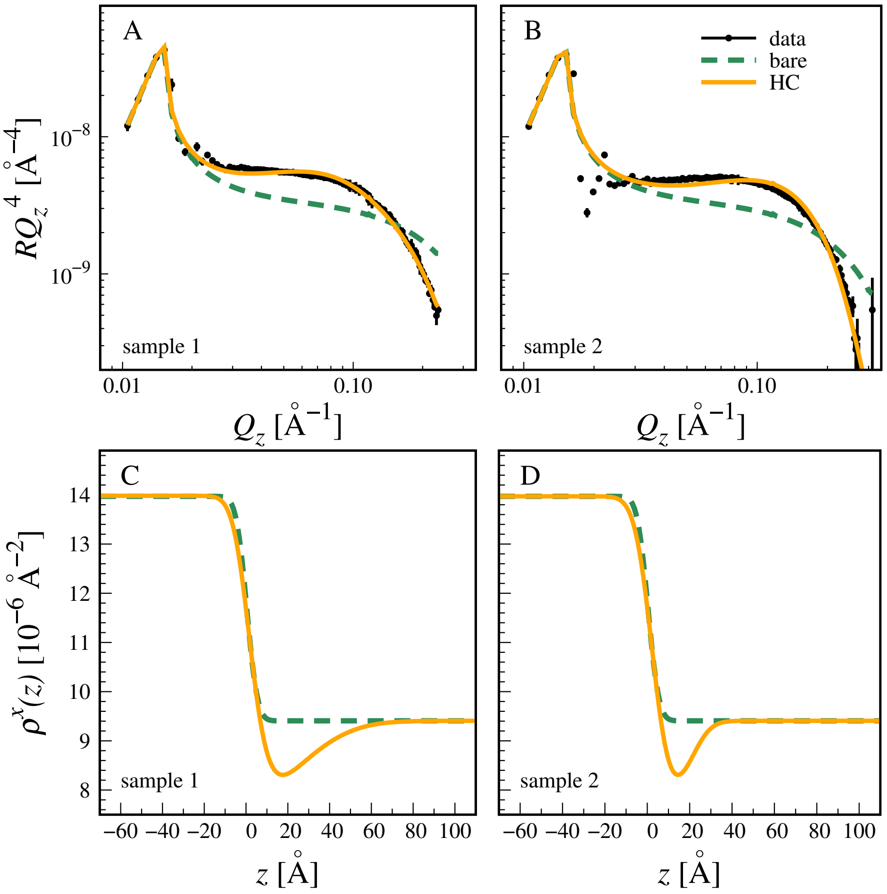

Figure SI.2: (A and B) Experimental x-ray reflectivity curves (symbols) of interfaces between water and PFO measured for sample 1 (A) and sample 2 (B). Dashed lines: theoretical reflectivity curves of the bare interface. Solid lines: theoretical reflectivity curves accounting for a distinct interfacial density deficit in the form of hydrocarbon chains (yellow line). (C and D) Associated interfacial profiles of the x-ray SLD.

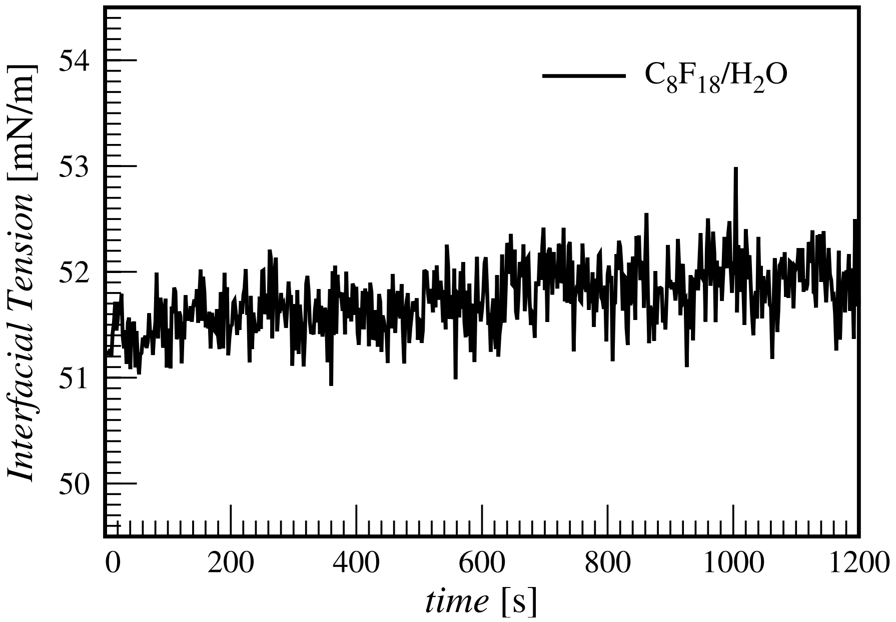

Figure SI.3: PFO/Water interfacial tension versus time for a C_8F_{18} drop in H_2O. Measurement was conducted using a PAT apparatus described at http://www.sinterface.de/pat1.html.
